# Factor structure and reliability of Persian version of hospital anxiety and depression scale in patients with breast cancer survivors

**DOI:** 10.1186/s12955-020-01429-6

**Published:** 2020-06-10

**Authors:** Karimollah Hajian-Tilaki, Erfaneh Hajian-Tilaki

**Affiliations:** 1 Department of Biostatistics and Epidemiology, Babol University of Medical Sciences, Babol, Iran; 2 Social Determinants of Health Research Center, Health Research Institute, Babol University of Medical Sciences, Babol, Iran; 3 Student Research Committee, Rasoul Psychiatric Hospital, Tehran University of Medical Sciences, Tehran, Iran

**Keywords:** Depression, Anxiety, HADS, Validity, Reliability, Persian, Breast cancer survivor

## Abstract

**Introduction:**

Anxiety and depression are significant concerns in breast cancer patients, and it may remain for a long term after primary treatments. The hospital anxiety and depression scale (HADS) is widely used to measure depressive and anxiety symptoms in clinical practices. The purpose of this study was to assess the psychometric properties of the Persian version of this scale in Iranian breast cancer survivors.

**Methods:**

A total of 305 patients with breast cancer, refered to Cancer Hospital in northen Iran and completed the primary treatments were enrolled in. All patients responded to a 14-item HADS. We performed confirmatory factor analysis (CFA) to examine the factor structure of HADS and the item-scale analysis in order to estimate the item reliability and consider the Cronbach’s alpha as a measure of internal consistency.

**Results:**

With a threshold of ≥8, the prevalence of anxiety and depression symptoms (moderate/severe) was 78.9 and 66.9%, respectively. The Cronbach’s alpha coefficients for anxiety and depression were 0.81 and 0.78, respectively. The CFA confirmed the two-factor structure model for HADS, indicating a good fitting summary indexes (χ2/df = 2.83, NFI = 0.88, RFI = 0.82, IFI = 0.92, CFI = 0.92, and RMSEA = 0.078).

**Conclusion:**

The CFA and item reliability analysis have indicated an excellent psychometric property of the Persian version of HADS to measure depressive and anxiety symptoms in breast cancer survivors. Thus, HADS is a useful screening tool to identify post-breast cancer anxiety and depressive disorders.

## Introduction

Anxiety and depression are the most common psychological disorders in breast cancer patients [1–4]. Patients are usually concerned about the effectiveness of used treatments, side effects of chemo-radiotherapy and their body shape images because of mastectomy. The psychological distress may stay for a long term among survivors. Besides, the fear of recurrence may be a more significant trigger for imposing an increase in anxiety and depression in women [1]. Several scales have been adapted to measure these psychological factors in breast cancer patients [5, 6]. Among the developed scales, the hospital anxiety and depression scale (HADS) containing 14 items is the most popular one used widely in clinical practices of psychiatric therapy in cancer patients.

Culture influences the way humans think about the environment and the world [7]. Although there are explicit criteria to ascertain anxiety and depression, culture also influences people to experience anxiety and depression. For example, the eastern culture emphasizes strong support for the group rather than individuals, it may produce stressors that provoke loss of individual identities and self-esteem and thus leads to depression and anxiety [8]. One problem of the use of HADS is a specific cutpoint may not be valid with cross-culture. Thus, the difficulties are increasingly being identified in discrepancies of optimal cutoff and factor structures of HADS.

This scale has been originally developed and validated by Zigmond and Snaith [9]. Recently, the HADS has been translated into several languages worldwide [10–15]. The validity and reliability of the Persian version of HADS used in breast cancer patients have not been adequately established. Despit, a preliminary validation of the Persian version of this scale was accomplished by Montazeri et al. [15] that had reported acceptable in reliability and validity measures. However, to assess the construct validity of this scale, they did not perform further details in item-scale analysis for item reliability and confirmatory factor analysis.

There is a debate in the literature about the number of factors that contribute to the construction of this scale for psychological distress in patients with cancer and other comorbidities [16, 17]. In a few studies, it was offered as a uni-dimensional factor [18], many others suggested as two- dimensional factors [9, 10, 19] and the others suggested the tri-dimensional [20–25] even four-factor model [10]. To validate the Persian version of HDAS, it is advisable to apply the HDAS at other stages of the cancer treatment not only after the primary treatment but also at different times of the cancer treatment and for the long term. Since the construction of the factors and magnitude of loading coefficients for this scale depend on disease conditions and social-culture status. Thus, the aim of this study was to validate the underlying structure of the HADS using confirmatory factor analysis (CFA) and to assess its reliability through item-scale analysis in Iranian breast cancer survivors.

## Methods and subjects

### Study design and participants

Data of this cross-sectional study were obtained from an educational hospital for cancer therapy, Shahid Rajaii, in the region of the north of Iran. We described this study as a secondary data analysis study. A total of 305 patients who were breast cancer survivors and completed the primary treatments were enrolled in the study from 1st January until the end of July in 2017. This referral cancer hospital has a broad coverage of the catchment population in the north of Iran. The details of the sample selection and characteristics of the study population were described elsewhere [26]. In brief, the patients who attended in periodic examination clinic were enrolled in the study. The prospective mean age (SD) of patients was 49.5 (10.1) years and also the mean (SD) duration from diagnosis was 3.5 (2.7) years. The Institutional Ethical Review Board of Babol University of Medical Sciences approved the study protocol (IRB code: IR.MUBABOL.HRI.REC.1398.361)

### Instrument and data collection

The HADS is a 14-item self-reported questionnaire that contains 7 items for anxiety and 7 items for depression [7, 8]. All items were rated on a 4-point Likert scale that shows how often patients suffer from anxiety and depressive disorders. Each item on the questionnaire is scored from 0 (not at all) to 3 (always), and this means that a person can score between 0 and 21 for either anxiety or depression. After transferring the score of reversed items in the uniform direction, in which the higher scores indicate a higher degree of anxiety and depression, the total score for each sub-scales of anxiety and depression can be ranged from 0 to 21, and thus; the total score is from 0 to 42. In the original scale, the score of 0–7, 8–15 and 16–21 indicate no/mild, moderate and severe depression/anxiety, respectively. Therefore, a cut-off point of 16 has been suggested for severe depression and anxiety as well. Additionally, in order to measure the quality of life (QoL) as a criterion of health outcome in breast cancer patients for assessment of HADS validity, the 30-item questionnaire, the European Organization for Research and Treatment of Cancer (EORT-C30) [27, 28] was simultaneously used in the current study. In the present study, a forward and backward translation of the HADS from English to Persian was done by two experts who were fluent in both English and Persian. The data collection was conducted in the waiting room at a periodical clinic with in-person interviews by trained nurses using HADS. Moreover, the demographic and clinical data were collected from all patients.

### Data analysis

A confirmatory factor analysis (CFA) was conducted using structural equation modeling (SEM) with a maximum likelihood method to estimate the factor structure and correlations. Before performing the analysis, the scores of reversed items were transferred in a way that the higher score shows a higher degree of severity in anxiety and depression. The single- and two-factor model was used. The single factor model was applied at two steps. Step one, no covariance or correlation was considered between observed indicators (Model 1-A), and step two, the correlations were established in a conceptual testing model (Model 1-B) between some relevant observed indicators. In performing the two-factor model, a correlation structure between the same observed indicators as model 1-B and between two factors were assumed (Model 2). The unifying structure of single-factor and two-factor models was assessed using the summary of fitting indices as χ2/df < 5, CFI > 0.90, RMSEA< 0.08 as a good fit [29]. Besides, in the present study, the comparative measure of fit such as the Akaike information criterion (AIC) was used to compare the two hypothesized models and to assess which model would fit better with observed data. The lower value of AIC is an indicator of a better fit in comparative conditions [30].

Additionally, the item-scale analysis was performed, and the internal consistency of HADS was evaluated using Cronbach’s alpha. Inter-item correlation and corrected-item total correlation were used to further assess construct and convergent validity. In addition, the item scale means, scale variance and Cronbach’s alpha if item deleted were used for assessment of the stability of the scale. Furthermore, the evidence of convergent and divergent validity of the HADS was shown by the pattern of the correlation of corresponded items of anxiety and depression. The total score of QoL for each individual was transferred from 0 to 100 and lower score indicated poorer QoL. Then, the QoL score was categorized as ≤50% or higher. The criterion validity of HADS was performed through estimating the discriminant power of HADS score in predicting the poor QoL using ROC analysis. The area under the curve (AUC), 95% confidence interval (95% CI), optimal cut-points for a total score of the HADS and its subscales were determined in the present study. Meanwhile, in order to identify the content validity, the correlation of item score with total HADS score and its subscales including QoL score were assesed. The SPSS software version 18.0 and AMOS software version 24.0 were used and the *p*-value less than 0.05 was considered as significant level.

## Results

### Socio-demographic characteristics of the study participants

The prospective mean age (SD) of participants was 49.5 (10.1) years. Table 1 illustrates the demographic and clinical characteristics of breast cancer patients. About 40 (13.1%) of the patients were under 40 years, 39 (12.8%) were 60 years or older, and the rest were 40–59 years. Roughly, in half of the participants, the duration (time since diagnosis) was ≥3 years. The education level of half of the cases was at the primary level or illiterate and 11.5% was academic.

**Table 1:** Demographic and clinical characteristics of study sample

Characteristics	n (%)
Age (year)	
< 30	4 (1.3)
30–39	36 (11.8)
40–49	109 (35.9)
50–59	116 (38.2)
> =60	39 (12.8)
Duration since diagnosis (year)	
< 3 y	155 (50.8)
3–5	78 (25.6)
> 5	72 (23.6)
Educational level	
Illiterate	71 (23.3)
Primary	80 (26.2)
Secondary/high school	119 (39.0)
University level	35 (11.5)
Anxiety	
Mild (0–7)	64 (21.1)
Moderate (8–15)	204 (67.3)
High (16–21)	35 (11.6)
Depression	
Mild (0–7)	100 (33.1)
Moderate (8–15)	190 (62.9)
High (16–21)	12 (4.0)
QoL	
Less than 50%	83 (28.0)
50% or higher	213 (72.0)

Values might not sum to *n* = 305 because of a few missing data from patients’ records

### Clinical characteristics

With a threshold of ≥8, the prevalence of anxiety and depression symptoms (moderate/severe) was and 78.9 and 66.9%, respectively. The majority of patients had an average level (total score 8–15) of anxiety (67.3%) and depression (62.9%), and the prevalence of high degree (total score ≥ 16) of anxiety and depression was 11.6 and 4.0%, respectively. The QoL of 83 (28.0%) of the participants was lower than 50% of the scale used (Table 1).

### Item-reliability analysis and reliability coefficient

In scale analysis, the reliability coefficients measured based on Cronbach’s alpha were 0.81 and 0.78 for anxiety and depression subscales, respectively. Table 2 demonstrates the mean (SD) scores of the items, scale means and scale variance if item deleted. Furthermore, the corrected item-total correlation and Cronbach’s alpha were estimated if item deleted according to anxiety and depression in scale analysis. The mean score of 4 items on anxiety was above the expected average of the scale (i.e. 1.5), while the mean score of items on depression was almost below the expected average. The mean scale for both factors had no significant change if item deleted. The corrected item-total correlations were rather low for item four (A4) in anxiety scale and for item four (D4) in depression scale. However, for both scales, the Cronbach’s alpha did not change sharply if item deleted, which indicates unifying the reliability of items in anxiety and depression scales. Meanwhile, the mean of the total score (SD) for anxiety and depression was 10.74 ± 4.17 and 8.93 ± 4.11, respectively.

**Table 2:** Item statistics and item –total statistics in scale analysis according to anxiety and depression scale

Items of HADS	Mean ± SD	Scale mean if item deleted	Scale variance if item deleted	Corrected item total correlation	Cronbach’s Alpha if item deleted
**Anxiety**					
A1. I feel tense or wound up	1.54 ± 0.89	9.02	13.07	0.55	0.78
A2. I get a sort of frightened feeling as if something bad is about to happen	1.63 ± 0.87	9.11	12.52	0.66	0.76
A3. Worrying thoughts go through my mind	1.66 ± 0.85	9.08	12.45	0.70	0.75
A4. I can sit at ease and feel relaxed	1.34 ± 0.89	9.40	15.85	0.11	0.85
A5. I get a sort of frightened feeling like butterflies in the stomach	1.65 ± 0.83	9.09	12.66	0.68	0.76
A6. I feel restless and have to be on the move	1.47 ± 0.87	9.27	12.42	0.69	0.75
A7. I get sudden feelings of panic	1.44 ± 0.91	9.30	13.42	0.47	0.79
Total score	10.74 ± 4.17	–	–	–	0.81
**Depression**					
D1. I still enjoy the things I used to enjoy	1.22 ± 0.87	7.71	12.36	0.62	0.73
D2. I can laugh and see the funny side of things	1.17 ± 0.86	7.76	11.91	0.72	0.71
D3. I feel cheerful	1.09 ± 0.90	7.83	12.67	0.54	0.75
D4. I feel as if I am slowed down	1.53 ± 0.92	7.40	14.68	0.20	0.81
D5. I have lost interest in my appearance	1.53 ± 0.92	7.40	13.91	0.32	0.79
D6. I look forward with enjoyment to things	1.18 ± 0.88	7.75	12.55	0.56	0.74
D7. I can enjoy a good book or radio or TV program	1.18 ± 0.88	7.75	12.25	0.64	0.73
Total score	8.93 ± 4.11	–	–	–	0.78

### Construct validity and factor structure

**Fig. 1:**
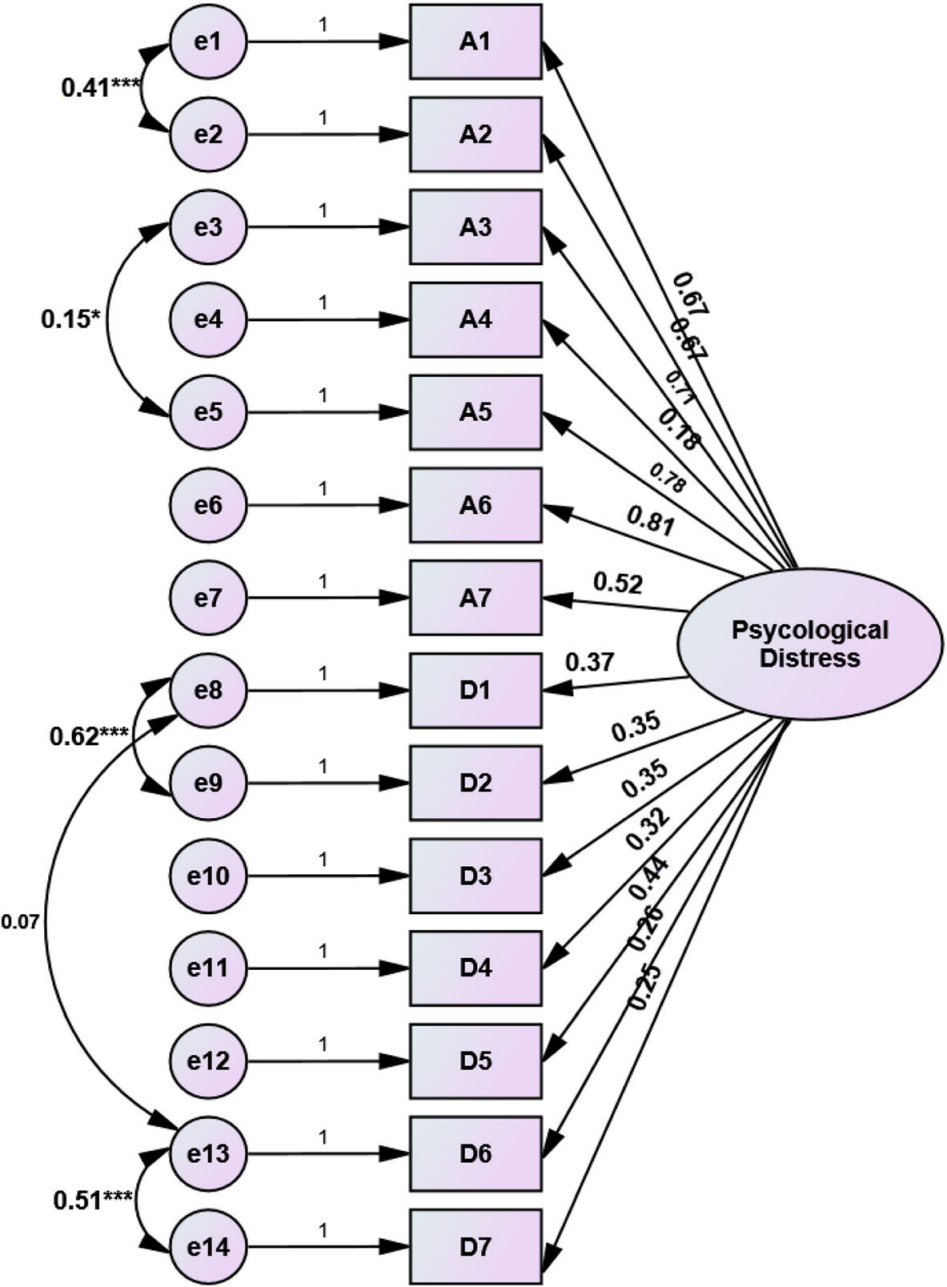
The structure of loading coefficients of a single factor model of the HADS and the correlations between some observed indicators (*p < 0.05; ***p < 0.001; the loading coefficients of all indicators were significant *p* < 0.001 and *p* < 0.01 for A4)

**Fig. 2:**
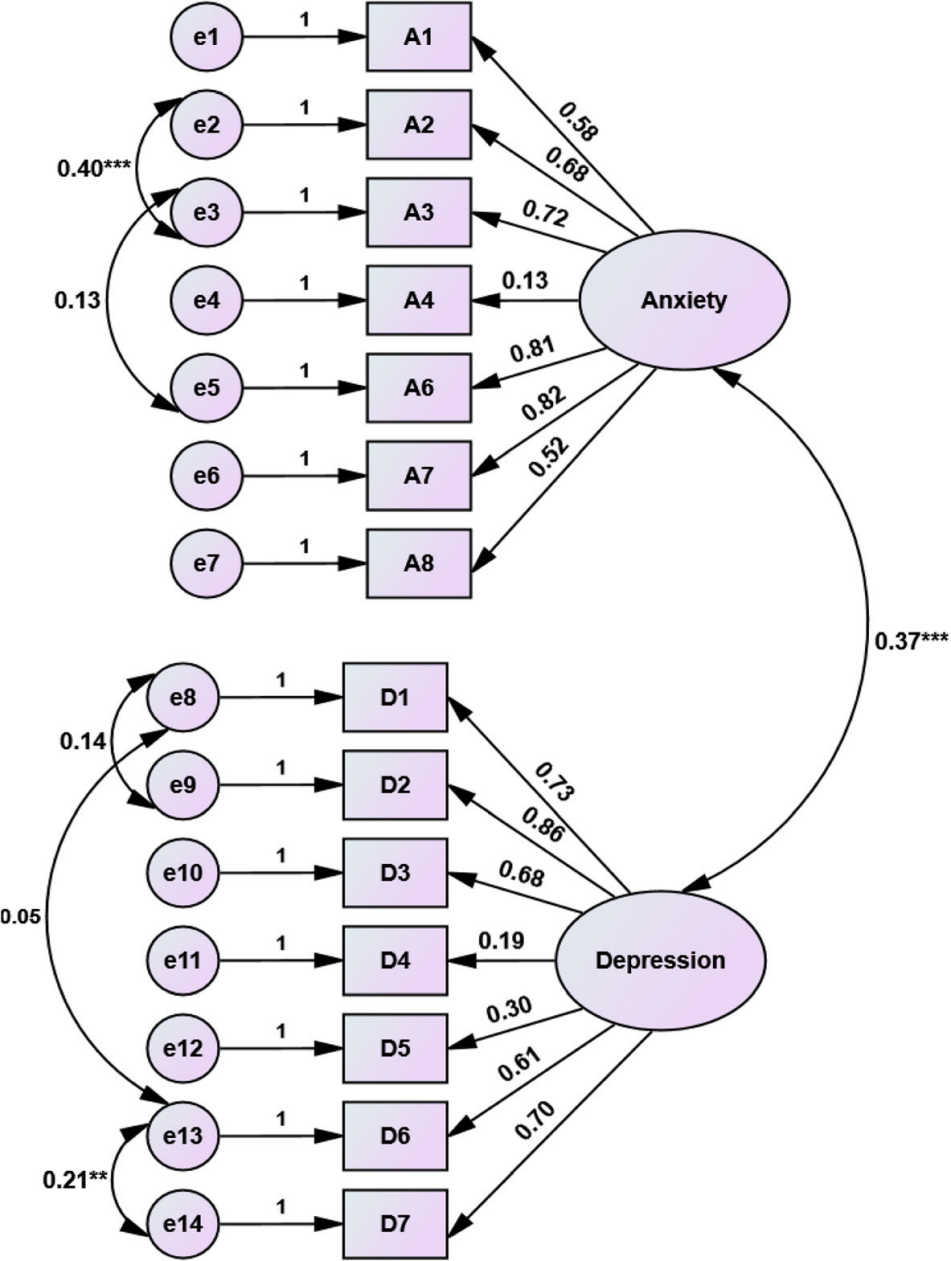
The structural loading coefficients of anxiety and depression and the correlation between two constructs and between some observed variables in CFA of two-factor model (**p < 0.01, ***p < 0.001, and *p* < 0.001 for all loading coefficients)

Figures 1 and 2 present the standardized loading factor coefficients of the single and two-factor models respectively. All loading coefficients were significant for both factor models (*p* = 0.001). In a single factor model, the greatest loading coefficient (beta = 0.81) was for item A6 and the lowest one (beta = 0.18) was for A4. Nevertheless, for the two-factor model, in the subscale of anxiety, the greatest impact of loading coefficient (beta = 0.82) was observed in 6th item (A6) and the lowest one (beta = 0.13) was for 4rth item. The item of D2 had the highest loading coefficient (beta = 0.86) for the depression factor. There was a significant correlation between the two factors of anxiety and depression (*r* = 0.37, *p* = 0.001). Table 3 represents that in comparison to single-factor models of 1-A and 1-B, the fitting criteria for model 1-B that conceived the correlations between some indicators (items) slightly improved. Additionally, both models of 1-A and 1-B as a single factor model had poor fitting performance, whearas the two-factor model with the establishment of the correlation structure between two factors and between some indicators indicated a good fitting summary index (NFI = 0.88, RFI = 0.82, IFI = 0.92, CFI = 0.92, RMSEA = 0.078, χ2/df = 2.83). Thus, the CFA confirmed the two-factor structure model for HADS.

**Table 3:** The summary fitting indexes of a single factor model and two factors model of the HADS

Model	NFI	RFI	IFI	CFI	PNFI	PCFI	RMSEA	χ^2^/df	*p*-value	AIC
Single Factor Model (1-A)	0.56	0.40	0.59	0.58	0.41	0.43	0.170	9.4	0.001	809.9
Single Factor Model (1-B}	0.74	0.62	0.77	0.77	0.51	0.53	0.129	6.04	0.001	528.67
Two- Factor Model	0.88	0.82	0.92	0.92	0.59	0.62	0.078	2.33	0.001	296.86

### Concurrent and criterion validity

Table 4 displays the spearman inter correlations between HADS items. An interesting finding in Table 4 was the inter-correlations within each subscale of depression and anxiety, which was significantly higher than the correlations of items between two subscales. These results were an indication of the convergent and divergent validity of HADS. Besides, in the assessment of criterion validity, our results suggested that higher score of HADS had the discriminant power to predict the low QoL (AUC = 0.82, 95%CI: 0.77–0.87). The discriminant accuracy of anxiety and depression subscales of HADS for prediction of low QoL was AUC = 0.80 (95%CI: 0.75–0.86) and 0.72 (95%CI: 0.65–0.77) with a cut-off point of 10 and 11, respectively. Table 5 shows a significant correlation between item scores in the subscale of anxiety and depression with corresponded total scores in scales and a total score of HADS. The item score in each subscale had a high correlation with the total score of the corresponded subscales and a lower correlation with other subscales. Additionally, a negative correlation between total scores of anxiety and depression with the score of QoL had been observed.

**Table 4:** Spearman’s correlations between items of HADS

	A1	A2	A3	A4	A5	A6	A7	D1	D2	D3	D4	D5	D6	D7
A1	1	0.46^∗ ∗ ∗^	0.46^∗ ∗ ∗^	0.10	0.42^∗ ∗ ∗^	0.47^∗ ∗ ∗^	0.39^∗ ∗ ∗^	0.25^∗ ∗ ∗^	0.21^∗ ∗ ∗^	0.17^∗∗^	0.20^∗ ∗ ∗^	0.30^∗ ∗ ∗^	0.15^∗∗^	0.13^∗^
A2		1	0.69^∗ ∗ ∗^	0.06	0.56^∗ ∗ ∗^	0.52^∗ ∗ ∗^	0.38^∗ ∗ ∗^	0.16^∗∗^	0.18^∗ ∗ ∗^	0.19^∗∗^	0.20^∗ ∗ ∗^	0.23^∗ ∗ ∗^	0.10	0.07
A3			1	0.12^∗^	0.61^∗ ∗ ∗^	0.56^∗ ∗ ∗^	0.37^∗ ∗ ∗^	0.24^∗ ∗ ∗^	0.22^∗ ∗ ∗^	0.23^∗ ∗ ∗^	0.21^∗ ∗ ∗^	0.29^∗ ∗ ∗^	0.12^∗^	0.15^∗∗^
A4				1	0.07	0.10	0.02	0.29^∗ ∗ ∗^	0.38^∗ ∗ ∗^	0.38^∗ ∗ ∗^	0.02	0.12^∗^	0.26^∗ ∗ ∗^	0.52^∗ ∗ ∗^
A5					1	0.69^∗ ∗ ∗^	0.38^∗ ∗ ∗^	0.21^∗ ∗ ∗^	0.22^∗ ∗ ∗^	0.23^∗ ∗ ∗^	0.24^∗ ∗ ∗^	0.32^∗ ∗ ∗^	0.10	0.10
A6						1	0.43^∗ ∗ ∗^	0.28^∗ ∗ ∗^	0.25^∗ ∗ ∗^	0.25^∗ ∗ ∗^	0.23^∗ ∗ ∗^	0.32^∗ ∗ ∗^	0.17^∗∗^	0.15^∗∗^
A7							1	0.05	0.07	0.05	0.26^∗ ∗ ∗^	0.27^∗ ∗ ∗^	0.03	−0.04
D1								1	0.69^∗ ∗ ∗^	0.49^∗ ∗ ∗^	0.14^∗∗^	0.14^∗∗^	0.49^∗ ∗ ∗^	0.49^∗ ∗ ∗^
D2									1	0.59^∗ ∗ ∗^	0.13^∗^	0.21^∗ ∗ ∗^	0.55^∗ ∗ ∗^	0.61^∗ ∗ ∗^
D3										1	0.04	0.15^∗ ∗ ∗^	0.40^∗ ∗ ∗^	0.50^∗ ∗ ∗^
D4											1	0.34^∗ ∗ ∗^	0.08	0.14^∗∗^
D5												1	0.21^∗ ∗ ∗^	0.22^∗ ∗ ∗^
D6													1	0.57^∗ ∗ ∗^
D7														1

*P < 0.05; ** *P* < 0.01; *** *P* < 0.001

**Table 5:** The inter -correlation between item score with total scores of anxiety, depression, total HADS and QoL score

Item score and Total scores	Anxiety score	Depression score	Total score of HADS	QoL score
A1	0.69***	0.28***	0.58***	−0.53***
A2	0.76***	0.22***	0.58***	−0.40***
A3	0.77***	0.30***	0.63***	−0.42***
A4	0.30***	0.39***	0.40***	−0.09
A5	0.76***	0.26***	0.59***	−0.44***
A6	0.78***	0.34***	0.66***	−0.51***
A7	0.63***	0.13*	0.47***	−0.54***
D1	0.31***	0.74***	0.67***	−0.26***
D2	0.32***	0.81***	0.58***	−0.31***
D3	0.30***	0.68***	0.35***	−0.22***
D4	0.27***	0.41 ***	0.51***	−0.26***
D5	0.39 ***	0.51***	0.55***	−0.34***
D6	0.19***	0.70***	0.82***	−0.24***
D7	0.17**	0.75***	0.81***	−0.20***
Anxiety score	1	0.44 ***	0.82***	−0.62***
Depression score		1	0.81***	−0.40 ***
Total score of HADS			1	−0.61***
QoL score				1

*p < 0.05, **p < 0.01, *** *p* < 0.001

## Discussion

The findings of the current study illustrated that the CFA confirmed the underlying structure of the two-factor model for the HADS. All related loading coefficients of observed indicators in the two-factor model were significant. All fitting indexes met the criteria for the goodness of fit for the two-factor model but not for a single factor model. Also, the scale item analysis revealed that the item-deleted reliability measures were ranged from 0.71 to 0.85, and a high-reliability coefficient of the overall score was observed for anxiety and depression. Several studies have explored the factor structure of the HADS in other languages [14, 16–24, 31]. Many researchers have support the two-factor structure [32–34], others have suggested a single factor model as a uni-dimensional measure of psychological distress [18] and some argue the three or four-factor structures for HADS [19–22]. These discrepancies of results in the literature are partially attributed to the exploratory factor analysis. Another explanation for the number of factors found in the structure of HADS may assign to the type of disease under investigation. Few studies were performed on CFA of HADS in cancer patients [10, 19], and many others were conducted on HADS in non-clinical settings or other chronic diseases [18–25]. Our findings regarding the factor structure of HADS are similar to those reported by Moorey et al. among cancer patients [19].

Based on current results, the total score of HADS and scores of its two subscales had an adequate discriminant power to predict the poor QoL in cancer patients. Moreover, it was explored that the cut points were 10 and 11 for subscales of anxiety and depression, respectively. The results also demostrated that a high item score correlated with the total score of HADS and the scores of its subscales, which is an indicator of concurrent validity. Meanwhile, different cut-off values for the HADS have been recommended in the literature [9, 10, 17, 35]. A meta-analysis explored the cut-off value of the total score of HADS, which varied from 10 to 15 depending on screening purposes (mental disorder or depressive disorder). In each subscale used, the threshold of 7 produced high sensitivity and specificity in four studies and the optimal threshold varied from 5 to 11 in different studies [17]. In another systematic review of a large number of studies, a cut-off point of 8 was offered for anxiety or depression [10]. In addition, in the original scale developed by Zigmond and Snaith, the cut-off point was 8 for depression and anxiety subscales [9]. In another study, a HADS total score of > 13 had sensitivities of 96% and specificities of 74% [35]. Nevertheless, the variation of cut-off values in different studies can be explained by the type of diseases and screening purposes as well as the objective definition of the used gold standard. One possible explanation for variations in the prevalence of psychological distress, especially anxiety and depression, is using different cut-off points in various studies. However, the ongoing study was not aimed to determine the optimal cut-off value for HADS because of the lack of implementation of the gold standard for assessment of psychological distress. Howsoever, in the present study, a proxy measure as self-reported QoL was established for evaluation of criterion validity.

Furthermore, we used the cut-point score of the original scale of 8 for moderate/severe psychological distress; the majority of our patients had mild depression and anxiety. In the current study, the prevalence of anxiety (moderate/severe) was higher than that of depression. Our findings in the prevalence of anxiety were in accordance with those reported by Montazeri et al. among Tehranian breast cancer women [36] while we found more moderate depression but the lower level of severe depression in comparison to Tehranian breast cancer. Additionally, a meta-analysis has demonstrated that the prevalence of depression was ranged from 14.0 to 95.9% and its pooled estimate was 46.8% among Iranian breast cancer patients [37]. Whereas the HADS identified 58.3% as depressed using the cutoff values of 8 among Italian cancer patients [38]. The differences may be attributed to variations in the clinical stage of the disease and/or socio-economic status and coping strategies used in psychotherapeutic management and cultural characteristics. The relatively high prevalence of moderate depression and anxiety in the current study highlights the need for psychological therapy of breast cancer patients along with standard treatment in the study region.

Based on the current study, the reliability coefficients of total score, measured based on Cronbach’s alpha were 0.81 and 0.78 for anxiety and depression, respectively. These reliability coefficients are similar to those reported in another study [15]. In our item-scale analysis, the scale means, scale variance and scale Cronbach’s alpha if item deleted had a negligible variation. This implies the necessity of all items for HADS. The corrected item-total correlation for the fourth item-- “I can sit at ease and feel relaxed”—in the subscale of depression was rather low. Perhaps, this might happen by error because the direction of the score of this item in terms of severity of anxiety was the opposite of other items in this subscale.

In terms of convergent and divergent validity, we used the correlation matrix of 14 items in HADS. The interesting findings of the current study were the correlation between items within subscales, which was higher than the items between the subscales. In other words, the items which measured the same characteristics were highly correlated, while the correlation of items that measure different constructs was lesser. This is an evidence of the convergent and divergent validity of the scale. Meanwhile, the findings of the ongoing study explored that the total score of HADS and its subscales for anxiety and depression had a good criterion validity in the prediction of QoL. However, the QoL as a proxy measure of criterion was used in the study to assess the criterion validity. We did not implement a gold standard for clinical measure, and our measure for evaluation of QoL was almost self-reported.

### Limitations and direction for future research

In this study, since the HADS may depend on the conditions of patients and underlying characteristics of the population, the adequacy of the two-factor model in explanation of the loading structure of HADS among breast cancer patients may not always be generalized. It would also be relevant to validate this version of the HDAS in different types of disease conditions. Another limitation was that we did not apply clinically further valid measures to assess the concurrent validity usually evaluated by other valid measures that are clinically acceptable such as Beck’s depression scale [39] and/or other anxiety and depressive inventory. Besides, in the present study, the QoL was used as a subjective measure to identify the criterion validity of the HADS. Although this study indicated that the Persian version of HADS obtained good validity and reliability, other studies should be conducted to confirm these characteristics in other clinical settings with other types of cancer. Thus, further studies are required to evaluate the discriminant predictive power of this scale in comparison with standard clinical measures among cross-ethnic groups of Iranian cancer patients.

## Conclusion

The two-factor model appeared to perform the best fit for the loading structure of HADS but not a single factor model, and our findings implied excellent psychometric properties of the Persian version of HADS in order to measure depressive and anxiety symptoms in breast cancer survivors. Thus, HADS is an effective screening tool to identify post-breast cancer anxiety and depressive disorders and to measure the impact of disease condition on depression and anxiety in Iranian breast cancer survivors.

## Data Availability

To keep patients’ confidentiality, the raw data would not be shared. But, it is available from the corresponding author on reasonable request, and the summary data are available in the main document.

## Abbreviations

HADS: Hospital anxiety and depression scale
CFA: Confirmatory factor analysis
SEM: Structural equation modeling
AIC: Akaike information criterion
*GFI*: *Goodness of* fit index
*NFI*: Normed fit index
*IFI*: Incremental fit index
*RMSEA*: *R*oot mean square error of approximation
CFI: Comparative fit index
PFI: Parsimony fit index
AGFI: Adjusted goodness of fit index
EORT-C30: 30 items -the European Organization for Research and Treatment of Cancer
ROC: Receiver operator characteristics
AUC: Area under the curve
SD: Standard deviation
CI: Confidence interval
A: Anxiety
D: Depression
QoL: Quality of life

## References

[1] Wei D, Liu X-Y, Chen YY, Zhou X, Hu HP (2016). Effectiveness of physical, psychological, social and spiritual intervention in breast cancer survivors: an integrative review. Pac J Oncol Nurs.

[2] Andrykowski MA, Lykins E, Floyd A (2008). Psychological health in cancer survivors. Semin Oncol Nurs.

[3] Yeh ML, Lee TY (2016). A prospective study of relationship between psychological factors and breasr cancer. Asia Pac J Oncol Nurs.

[4] Falagas ME, Zarkadoulia EA, Ionnidan EN, Peppas G, Christodonlon C, Rafailidis PI (2007). The effect of psychological factors on breast cancer outcome: a systematic review breast. Cancer Res.

[5] Aukst-Margetic B, Jakovljevic M, Margetic B, Peppas G, Christodoulon C, Rafailidis PI (2005). Religiosity, depression and pain in patients with cancer. Gen Hosp Psychiatry.

[6] Beck AT, Steer RA, Garbin MGJ (1988). Psychometric properties of the Beck depression inventory twenty-five years of evaluation. Clin Psychol Rev.

[7] Noguchi K (2014). Differences in the primary effect for person perception. Int J Chycol.

[8] Sangalang CC, Gee GC (2012). Depression and anxiety among Asian American: the effects of the social support and strain. Soc Work.

[9] Zigmond AS, Snaith RP (1983). The hospital anxiety and depression scale. Acta Psychiatr Scand.

[10] Bjelland I, Dahl AA, Haug TT, Neckelmann D (2002). The validity of the hospital anxiety and depression scale. An updated literature review. J Psychosom Res.

[11] Reda AA (2011). Reliability and validity of the Ethiopian version of the Hospital Anxiety and Depression Scale (HADS) in HIV Infected Patients. PLoS ONE.

[12] Bocéréan C, Dupret E (2014). A validation study of the hospital anxiety and depression scale (HADS) in a large sample of French employees. BMC Psychiat.

[13] Miklavcic IV, Snoj Z, Mlakar J, Pregelj P (2008). Validation of the Slovenian version of Hospital Anxiety and Depression Scale in female cancer patients. Psychiatr Danub.

[14] Barth J, Martin CR (2005). Factor structure of the hospital anxiety and depression scale (HADS) in German coronary heart disease patients. Health Qual Life Outcomes.

[15] Montazeri A, Vahdanian M, Ebrahimi M, Jarvandi S (2003). The hospital anxiety and depression scale (HADS): translation and validation study of the Iranian version. Health Qual Life Outcomes.

[16] Cosco TD, Doyle F, Ward M, McGee H (2012). Latent structure of the hospital anxiety and depression scale: a 10-year systematic review. J Psychosom Res.

[17] Vodermaier A, Millman RD (2011). Accuracy of the hospital anxiety and depression scale as a screening tool in cancer patients: a systematic review and meta-analysis. Support Care Cancer.

[18] Razavi D, Delvaux N, Farvacques C, Robaye E (1990). Screening for adjustment disorders and major depressive disorders in cancer in-patients. Br J Psychiatry.

[19] Moorey S, Greer S, Watson M, Gorman C, Rowden L, Tunmore R, Robertson B (1991). The factor structure and factor stability of the hospital anxiety and depression scale in patients with cancer. Br J Psychiatry.

[20] Clark LA, Watson D (1991). Tripartite model of anxiety and depression: psychometric evidence and taxonomic implications. J Abnorm Psychol.

[21] Joiner T (1996). A confirmatory factor-analytic investigation of the tripartite model of depression and anxiety in college students. Cogn Ther Res.

[22] Joiner TE, Catanzaro SJ, Laurent J (1996). Tripartite structure of positive and negative affect, depression, and anxiety in child and adolescent psychiatric inpatients. J Abnorm Psychol.

[23] Caci H, Bayle FJ, Mattei V, Dossios C, Robert P, Boyer P (2003). How does the hospital and anxiety and depression scale measure anxiety and depression in healthy subjects?. Psychiatry Res.

[24] Friedman S, Samuelian JC, Lancrenon S, Even C, Chiarelli P (2001). Three-dimensional structure of the hospital anxiety and depression scale in a large French primary care population suffering from major depression. Psychiatry Res.

[25] Amini P, Maroufizadeh S, Omani SR (2017). Evaluating the factor structure, item analyses, and internal consistency of hospital anxiety and depression scale in Iranian infertile patients. IJRM..

[26] Firouzbakht M, Hajian-Tilaki K, Moslemi D. Analysis of quality of life in breast Cancer survivors using structural equation modeling: the role of spirituality, social support, and psychological well-being. Int Health. 2019. 10.1093/inthealth/ihz108. 10.1093/inthealth/ihz108 PMC7322199 31927594

[27] Montazeri A, Harichi I, Vahdani M (2000). The EORTC breast cancer-specific quality of life questionnaire (EORTC QLQ-BR23): translation and validation study of the Iranian version. Qual Life Res.

[28] Aaronson NK, Ahmedzai S, Bergman B (1992). The European Organization for Research and Treatment of Cancer QLQ-C30: a quality-of-life instrument for use in international clinical trials in oncology. J Natl Cancer Inst.

[29] Hooper D, Coughlan J, Mullen MR. Structural equation modelling: guidelines for determining model fit. Electronic J Business Res Methods. 2008;2.

[30] Akaike H (1974). A new look at the statistical model identification. IEEE Trans Autom Control.

[31] López PM, Ferrandis ED, Vaillo YW, Garrido MJG, Pérez SM, Guerra EI (2012). Structural validity and distress screening potential of the hospital anxiety and depression scale in cancer. Int J Clin Health Psychol.

[32] Hung M, Bounsanga J, Tang P, Chen W, Cheng C (2015). The factor structure of the hospital anxiety and depression scale in orthopedic trauma patients. J Clin Med Res.

[33] Mykletun A, Stordal E, Dahl AA (2001). Hospital anxiety and depression (HAD) scale: factor structure, item analyses and internal consistency in a large population. Br J Psychiatry.

[34] Annunziata MA, Muzzatti B, Altoè G (2011). Defining hospital anxiety and depression scale (HADS) structure by confirmatory factor analysis: a contribution to validation for oncological settings. Ann Oncol.

[35] Alexander S, Palmer C, Stone PC (2010). Evaluation of screening instruments for depression and anxiety in breast cancer survivors. Breast Cancer Res Treat.

[36] Montazeri A, Harirchi I, Vahdani M (2000). Anxiety and depression in Iranian breast cancer patients before and after diagnosis. Euro J Cancer Care.

[37] Ahmadi Gharaei H, Dianati Nasab M, Kouhestani SM, Fararouei M, Moameri H, Pakzad R, Ghaiasvand R (2019). Meta-analysis of high prevalence of depression among breast cancer survivors in Iran: calling community supportive care programs. Epidemiol Health.

[38] Castelli L, Binaschi L, Caldera P, Mussa A (2011). Fast screening of depression in cancer patients: the effectiveness of the HADS. Euro J Cancer Care..

[39] Beck AT, Epstein N, Brown G, Steer RA (1988). An inventory for measuring clinical anxiety: psychometric properties. J Consult Clin Psychol.

